# Objective and subjective career success: individual, structural, and behavioral determinants on European hybrid workers

**DOI:** 10.3389/fpsyg.2023.1161015

**Published:** 2023-06-20

**Authors:** Kiall Hildred, Margarida Piteira, Sara Cervai, Joana Carneiro Pinto

**Affiliations:** ^1^Universidade Católica Portuguesa, Católica Research Centre for Psychological, Family and Social Wellbeing, Lisbon, Portugal; ^2^ISCAL, Lisbon, Portugal; ^3^Department of Social and Political Sciences, University of Trieste, Trieste, Italy

**Keywords:** objective success, subjective success, hybrid workers, individual, structural and behavioral determinants

## Abstract

**Introduction:**

In the current worldwide labor context, where a disruption took place and employees experience.

**Methods:**

Participated in this study 739 European hybrid workers who fulfilled an online assessment protocol.

**Results:**

Results indicate that higher ages, higher educational levels, being married, having children, working.

**Discussion:**

This study makes a unique contribution to the extant research on hybrid workers’ careers, specifically.

## Introduction

The goal of this study is to investigate the effects of a set of individual, structural and behavioral determinants on the objective and subjective career success of a sample of European hybrid workers.

Career success is an unquestionably relevant issue for individuals, organizations, and societies (Ng et al., 2005; Sullivan and Baruch, 2009), as employees’ personal success is often associated with organizational success and, ultimately, also contributes to societal success and well-being (Dyke and Murphy, 2006).

The definition of career success has become increasingly individual, varying from person to person throughout their lifespan, and depending on the existing personal, organizational, and social circumstances. In addition to its individual dimension, the definition also has a comparative dimension. That is, being successful implies reaching one’s goals and assessing the extent to which doing so has helped fulfil important needs (Baruch, 2004, p. 76), but it also implies assessing one’s position on the broader “social ladder” (Ng et al., 2005).

In the literature, career success is often defined as the set of positive outcomes, both professional and psychological, that a person has achieved throughout their working life (e.g., Seibert and Kraimer, 2001; Ng et al., 2005; Breland et al., 2007). This definition includes both objective (or extrinsic) components, such as position, salary and promotions, and subjective (or intrinsic) components that include the enjoyment, satisfaction and well-being that result from aspects of work such as work-family balance and perceived financial security (Dries et al., 2009; Mohd Rasdi et al., 2011; Seibert et al., 2013; Shockley et al., 2016). Therefore, these constitute two different components of success, with different conceptual understandings and, consequently, different evaluation methods (Abele and Spurk, 2009). However, most studies indicate some weak to moderate interdependence between them (e.g., Dette et al., 2004; Arthur et al., 2005; Hall and Chandler, 2005; Ng et al., 2005; Abele et al., 2011). For example, there is a strong possibility that a person with a higher salary and position feels more fulfilled and professionally satisfied, increasing their subjective success, while that same objective success could lead them to feeling more pressured to continue to perform well, decreasing their subjective success. In this regard, the two dimensions of success may not always be congruent.

Over the past few years, the literature on career success has proliferated, and has attempted to identify a set of determinants that may promote or hinder attainment of that success (e.g., Boudreau et al., 2001; Seibert and Kraimer, 2001; Lortie-Lussier and Rinfret, 2005). Numerous studies have looked at a wide range of individual (e.g., gender, age, educational degree, marital status, children), structural (e.g., organizational support and socialization) and behavioral (e.g., personality traits, self-efficacy, career identity) factors as potential determinants of success (Briscoe et al., 2015; Spurk et al., 2019). In general, it appears that objective and subjective career success do not necessarily have the same predictors (Spurk et al., 2019). However, it should be noted that most studies have been developed with employees who are on more traditional career trajectories, i.e., those who have worked within one or two organizations throughout their entire working life and on linear, rigid, unidirectional paths with a degree of predictability. In this context, seniority, loyalty, and commitment to the organization are often rewarded with objective success, namely with salary, promotions, and increasingly higher positions (Moen and Roehling, 2005). In contrast, in the new intelligent (e.g., DeFillippi and Arthur, 1994), protean (Briscoe and Hall, 2006), and boundaryless (Sullivan and Arthur, 2006) career paths – which are multidirectional, flexible and dynamic – each person manages their career according to their specific needs, goals and priorities. That is, career success acquires a more subjective dimension in which a greater sense of connection, interdependence, happiness and well-being at work are preferred (e.g., Harrington and Hall, 2007). Also, due to the recent COVID-19 pandemic, career patterns have been changing even more significantly, and much of what we know about them and about (managing careers in) the organizational contexts may no longer apply. A large proportion of workers throughout Europe now carry out their professional activities mostly (or almost exclusively) from their homes. This situation has repercussions not only in the way people work, but also in the way they perceive and manage their career success. In this sense, it is crucial to explore the impact of these variables – individual (personal and family), organizational/structural and behavioral – on career success in the context of new professional realities, namely hybrid work, given its increasing prevalence following the COVID-19 pandemic.

Before the pandemic, only a small percentage of people did their work remotely or in a hybrid way. However, the pandemic precipitated the use of remote and hybrid work as an organizational practice. At the European level, before the pandemic, about 15% of workers used computers, phones, and digital platforms to do their work off-site at their employer’s or a client’s premises (EuroFound, 2021). This type of work was mainly implemented informally, based on reliance on ICT, and access to a shared computer network, and was performed mostly by young male workers who were fairly skilled specialists. After the pandemic, the practice of remote and hybrid work became widespread. According to the EuroFound survey (2021), at the European level, approximately 40% of workers were in these working arrangements, with countries like Spain, Greece, Poland being slightly below the European average (about 30%), countries like France, Portugal, Italy being very close to the European average (between 40 and 45%) and countries like Luxembourg, the Netherlands and Finland, exceeding this number (about 58–60%). The pandemic came with a change of mentality and stigmas related to remote and hybrid working among companies and workers, and accelerated its massification (Bartik et al., 2020). Even nowadays, data from Eurostat and Statista reveals that, on average, across the European Union, 30% of workers regularly work from home (either fully remotely or in a hybrid model; Eurostat, Statista, 2022). It is important that we, as a society, help people manage their careers (professional lives) in an organizational world that, after an initial distrust of remote and hybrid work, is beginning to see this experience as potentially very advantageous. In this study, hybrid work refers to a working arrangement in which, at least a few days per week, work is performed by means of and/or with the aid of telecommunications technology and replaces (home-work) commuting.

## Theoretical framework and research design

Building on the work by Spurk et al. (2019) about antecedents and outcomes of objective and subjective career success, in which they concluded that “the full range and prevalence of theoretical approaches to the study of career success, and the extent to which different theoretical approaches have been conceptually and empirically compared and contested in past research, remains nuclear (p. 4),” the present paper does not consider any overarching theory. The theoretical framework of this study comprises a set of personal, family, structural/organizational and behavioral determinants of objective and subjective career success, which are analyzed below with respect to the literature.

### Personal variables

#### Gender

Gender is critical to the determination of career success and is present in a wide range of studies (e.g., Judge et al., 1995; Nabi, 2001; Ng et al., 2005; Volmer and Spurk, 2011; Ng and Feldman, 2014; Agrawal and Singh, 2021; Hofmann et al., 2021; Sönmez et al., 2021). Women, even if they have comparable levels of education to men, are often associated with lower salaries, lower-level positions in the corporate hierarchy, and fewer opportunities for career development and advancement, compared to their male counterparts (Evers and Sieverding, 2014; Sato et al., 2019). Companies justify these findings by stating that women, regardless of their education, are always more involved in, and responsible for, child-and home-care and, as a result, they invest less on their education and training, are less productive, work fewer hours, take more career breaks or withdraw from their jobs earlier than men (Mainiero and Sullivan, 2006; Valcour and Ladge, 2008). Thus, gender is negatively associated with objective success and affects subjective success through that association (Valcour and Ladge, 2008).

#### Academic degree

In addition to gender, a relationship also exists between academic degree and career success, particularly objective success (Judge et al., 1995; Nabi, 1999; Ng et al., 2005). The higher the academic degree, the more likely the person is to get a job that pays better, with more possibilities of advancement, and of accessing higher levels of hierarchy and greater status, autonomy, responsibility and power. These characteristics may, in turn, lead the person to feel greater pleasure, satisfaction and happiness with their professional situation or, on the contrary, and as previously mentioned, be considered a burden (Hall and Chandler, 2005). In this sense, studies on the influence of academic degree on success are not unanimous in their conclusions.

#### Age

For many studies on career success and development, age has been predominantly used as a control variable, or for purely descriptive purposes, and not as a central point of investigation (e.g., Volmer and Spurk, 2011; Evers and Sieverding, 2014; Zacher, 2014; Jung and Takeuchi, 2016; Sato et al., 2019; Smale et al., 2019; Sönmez et al., 2021; Kim and Kim, 2022). Hence, the findings on age and its influence on different outcomes in career development and success vary widely. These effects seem to occur at later ages, once relative ages among colleagues become apparent or when individuals reach ages for which age norms affect how they and others perceive their career progress and success (Lawrence, 1996). This can be seen in the difficulties older workers have, particularly women, in trying to re-enter the workforce (Cabrera, 2007). Yet these same age norms can also affect younger workers, who may not be perceived as ready for certain levels of career progress, or not feel experienced enough to have earned some level of success when comparing themselves to older colleagues.

The effects of age on career success also seem to differ between genders. While age has been found to be a significant predictor of subjective career success (as well as both career and job satisfaction) regardless of gender (Lortie-Lussier and Rinfret, 2005), there is evidence of differences in the influence of age between men and those women who have children. For women with children, the age at which they have children, and the stage in their career in which they have them, can significantly affect their career development and outcomes (Valcour and Ladge, 2008). Yet, this only seems to be the case for objective measures of success. What matters for the subjective career success of women with children is how long it has been since they had a child. This suggests that the growing capacity to balance work and life priorities as children grow older, and perhaps also the organizational and leadership skills parents can develop in the course of raising children, lead to greater subjective career success, whereas salaries and promotions are determined mostly by time spent working and are hence negatively affected by the interruption that having children creates.

In light of these findings, we propose the following hypotheses regarding personal variables and objective and subjective career success:

*H1*: Being male and educated will have a positive impact on achieving career success.

*H2*: Being female (even if educated), will have a negative impact on achieving career success (both objective and subjective).

*H3*: Age will have a positive effect on both – objective and subjective career success.

### Family variables

#### Marital status and children

Regarding family status, previous studies have consistently pointed to the widespread belief that being married is an indicator of stability and responsibility, though only for men (Judge et al., 1995; Stroh et al., 1996). In women, being married, and particularly having a family with one or more children, is perceived as a detrimental factor to career success. Women who are mothers are judged as less productive, competent or committed in their careers than men or those women without children (Budig and England, 2001; Correll et al., 2007). Furthermore, as previously noted, they are more likely to interrupt their careers during a period critical to their establishment and advancement, which subsequently has a lasting effect on salary and promotions (Lyness and Thompson, 1997; Kirchmeyer, 1998; Ziefle, 2004). Moreover, due to the increased responsibilities at home associated with parenthood, having children is also associated with lower levels of subjective success, particularly regarding work-family balance, and this increases the burden on women (O'Neil et al., 2008; Evers and Sieverding, 2014). All these circumstances lead to lower investment in workers with children, especially if they are women, and to greater discrimination by peers, superiors, and potential employers (Valcour and Ladge, 2008).

Hence, it is proposed that:

*H4*: Being a single man will have a positive impact on achieving career success, particularly objective success.

*H5*: Having children (for both men and women) will have a negative impact on achieving both objective and subjective career success.

### Structural variables

#### Size of the organization

There are good reasons to suggest that any effect of company size on career outcomes is entirely mediated by other variables (Kalleberg and Van Buren, 1996), and the findings on the relationship between company size and objective and subjective success have been mixed.

Some research has suggested a positive relationship between company size and objective career success (Cox and Harquail, 1991; Nabi, 1999), with Ng et al. (2005) finding mild support for a positive relationship between organizational resources (operationalized as size of organization) and career success. Whereas others have found negative relationships, such as Judge et al. (1995), who found a significant but small negative relationship between size and salary, and Cox and Nkomo (1991) who found a negative relationship between organization size and promotions. However, while Orser and Leck (2010) did find a positive relationship between organization size and hierarchical promotions, they failed to find one between size and salary.

The size of the organization also influences the levels of success achieved by employees, particularly with regard to objective success. That is, larger organizations are perceived as having greater growth potential and, consequently, greater ability to enable opportunities for career advancement (Nabi, 1999).

At least one study suggests that this perception also has an indirect effect on subjective success via the perception of objective success, i.e., employees who perceive that their organizations offer more opportunities for advancement, status and power, may feel prouder of their achievements and more satisfied with their future career prospects, thus reporting higher levels of subjective success (Bozionelos, 1996).

Other results regarding subjective career success are similarly mixed, with some studies suggesting a relationship with organizational size (Judge et al., 1995), and others failing to find support for any link (Nabi, 1999). While Yeh et al. (2018) found evidence to suggest that subjective career success would be lower in larger companies, as individuals in these companies report high work demands and burnout, many more studies have linked subjective career success to the greater senses of security and career outlook that can be provided in larger organizations (Wagner, 1997; Anderson and Pontusson, 2007).

These findings lead us to hypothesize that:

*H6*: There will be a positive relationship between company size and both objective and subjective career success.

#### Company type

The main implication for company type on career success outcomes is that incomes in the public sector are far more controlled than those in the private sector and do not tend to reach the same level of compensation for top-level executives (Orser and Leck, 2010). For subjective career success, however, the findings are not so straightforward. There is some reason to suggest that the working environment in the private sector leads to higher levels of burnout, where employees work longer hours than those in the public sector, and where there are weaker work-life boundaries and higher expectations for after-hours training and development (Fernando and Cohen, 2013; Yeh et al., 2018), suggesting that the psychological toll may affect subjective career success and career satisfaction.

However, there may also be a self-selection bias in the subjective levels of career success and career satisfaction. Public and private organizations may specifically attract people with personal measures of subjective career success that align with the environment and mission of those sectors. If those whose subjective career success and career satisfaction align with objective career measures, they may be attracted more by private organizations, where a greater potential for high salaries and promotions is available, whereas those whose measures concern social impact may be more inclined toward public organizations, where social impact is the purpose. In the extreme case, no differences in subjective career success would exist despite differences in objective career success.

This tracks well with Abele et al. (2011), who found a tighter relationship between objective and subjective success in the private sector than in the public sector, but it would contradict Al-Hussami et al. (2018) who found that those in the public sector had significantly higher levels of subjective career success than those in the private sector.

One further confounding variable in this relationship is gender. There is reason to suggest that levels of career success may vary for men and women differently within the private and public sectors. Differences in the distribution of genders among top-level positions within public and private organizations – with more women in higher levels of organizational hierarchy in public sectors than in private sectors (Orser and Leck, 2010; Ashworth et al., 2022) – may lead to differences between the sectors in the variability of career success outcomes between genders.

However, as Lortie-Lussier and Rinfret (2005) note, there are many studies that show no differences in career satisfaction despite differences in objective career success measures, regardless of sector (Kirchmeyer, 1998; Tharenou, 1999; Lemire and Saba, 2002). However, the studies cited are now more than 20 years old, and these levels may have been significantly affected by more recent developments in gender equality and the nature of work.

In light of these findings, it is hypothesized that:

*H7a*: There will be a positive relationship between working in the private sector and objective career success measures.

*H7b*: There will be no difference in the effect working in public or private organizations has on subjective career success.

#### Employment status

The career success literature has largely ignored non-full-time workers in its analyses (Valcour and Ladge, 2008). This is partly due to the assumption that employment status is an indicator of objective career success, or part of the definition of it (Nicholson, 2000; Heslin, 2005). As such, there is little available research investigating the direct effects of employment status (full-time/part-time) on career success outcomes. Most findings are of mediated or indirect effects. The study by Valcour and Ladge (2008) did investigate a direct link, finding that more time spent in part-time employment meant lower income, hence showing a link between employment status and objective career success, but they did not find a relationship between the amount of time spent in part-time employment and subjective career success.

There are some studies available linking employment status to subjective career success, such as that by Allen (2011) on nurses, which showed less career satisfaction among “casuals” than permanent employees. Yet, these conceptual distinctions between full-time/part-time and between permanent/casual are not identical – on can work full-time hours on a casual status, or have permanent status while working a casual schedule. Similarly, Lee and Johnson (1991) found that full-time workers had lower job satisfaction than part-time workers when they were temporary, but found no significant differences between full-and part-time workers when those workers were permanent.

The greatest impact part-time work may have on career success outcomes is in the negative perceptions toward part-time workers in regards to their career commitment, particularly for women who take on part-time work in order to have time available to care for their children (Cabrera, 2007; McDonald et al., 2008; Valcour and Ladge, 2008; Benschop et al., 2013; Kmec et al., 2014).

These findings suggest that employment status may have an effect on objective career success, due to its relation to income, but only limited effects on subjective career success. Hence, we propose the following hypotheses:

*H8a*: Part-time workers will score lower on measures of objective career success than full-time workers.

*H8b*: There will be no difference in the effect that working part-or full-time will have on subjective career success.

### Behavioral variables

#### Strategic career behaviors

Over the past few years, the changes that occurred in the labor market have made careers more volatile, unpredictable, and dynamic (Arnold, 1997), which requires employees to constantly acquire, develop, and apply strategic career behaviors (Sullivan and Mainiero, 2008; Greenhaus et al., 2010; Pinto, 2010). In this study, we considered three strategic career behaviors that comprise the Kaleidoscope Career Model (KCM; Sullivan and Mainiero, 2008): authenticity, balance and challenge. Authenticity refers to seeking alignment between organizational and personal values, balance refers to seeking equilibrium between professional and non-professional responsibilities, and challenge refers to seeking stimulation at work and career progression opportunities.

Previous studies have used the KCM to investigate various relationships between career-relevant factors. Mainiero and Sullivan (2005) used it to propose a more complex understanding of the trend of women leaving the labor market, and Cabrera (2007) used it to investigate the changes in career focus among women as they transitioned from early to late stages in their careers. It has been used by Grady and McCarthy (2008) to understand how professionals in mid-career stages balance work and life, and by Kirk (2016) to understand the differences in career stage timing and the type of work responsibilities assigned by managers. Kuzhabekova and Lee (2018) used it to look at why some academics become self-initiated expatriates. Mainiero and Gibson (2018) investigated the importance of each KCM dimension for men and women over time, and, similarly, O’Neill and Jepsen (2019) examined the dimensions in relation to various life roles. O’Connor and Crowley-Henry (2020) used it to show that the underemployment of skilled migrants is largely due to choices made by those migrants in the interests of balance.

However, while Koekemoer and Crafford (2019) investigated qualitative links between the KCM dimensions and subjective career success, the only study that has looked at a direct, quantitative link between the KCM dimensions and both objective and subjective career success outcomes was conducted by Simmons et al. (2022). This was conducted over a seven-year period to account for the changing nature of careers as considered by the KCM. The authors found that authenticity was not a significant predictor of career outcomes, but that changes in authenticity over time had a negative effect on career satisfaction. Furthermore, they found that balance significantly predicted salary, though the relationship was negative, but that balance did not significantly predict promotions or career satisfaction. It was also not a significant predictor of promotion rate, yet change in balance over time was, in the positive direction. Lastly, they did not find challenge to significantly predict any career outcomes, but they did find changes in challenge over time to significantly predict salary, promotions, promotion rate, and career satisfaction, all in the positive direction.

Based on these findings, we propose the following hypotheses:

*H9a*: Authenticity will not be a predictor of objective career success, but a negative predictor of subjective career success.

*H9b*: Balance will negatively predict objective career success, but will not be a predictor of subjective career success.

*H9c*: Challenge will positively predict both objective and subjective career success.

## Methods

The present methodological strategy follows the hypothetical-deductive assumptions based on the previously presented theoretical framework. Due to the nature of the current research problem, this is an exploratory study.

### Participants

A total of 739 European hybrid workers participated in this study (women = 283, 38.3%), with a mean age of 27.64 years old (SD = 8.48; Min = 18; Max = 70). About 32% (*n* = 236) of the participants were residents in Portugal, 26% (*n* = 192) in Poland, 11% (*n* = 80) in Italy, and 7% (*n* = 50) in Greece. Of these participants, 195 (26.4%) were married and 639 (86.5%) had no children. In terms of academic degree, 275 (37.2%) had a secondary diploma, 301 (40.7%) had a bachelor, and 163 (22.1%) had a master or doctorate degree. Participants were working for European employers (*n* = 739, 100%), predominantly full-time (*n* = 398, 53.9%), in private companies (*n* = 550, 74.4%), and in the media, culture and graphical (*n* = 116, 14.7%); mechanical and electrical engineering (*n* = 90, 11.4%); commerce (*n* = 84, 10.6%); and education (*n* = 79, 10%) sectors. The percentage of work they currently do in a hybrid situation is about 46.35% on average. 28.7% of the participants work from home or another location other than the company’s context about 2 to 3 times per week and 26% indicate doing it daily. In terms of weekly work hours, 27.5% of the participants perform about 21 to 40 h per week of remote work. The average salary of these participants is €1177.95 (*sd* = €835.23; Min-Max = 500–5,000). 30.8% of participants received on average €1249.50 monthly and 17.9% received on average €1999.50. 7% of the participants considered their salary to be within the average salary received for their current position in their country. About 40% of the participants have seen their salary increase between 10 and 25% in the last 6 years and 35% of the participants have been promoted between 1 and 2 times in the same period.

### Instruments

*Individual variables* included gender (1 = male; 2 = female), age (organized in four age-range groups corresponding to early adult transition/pre-adulthood) [1 = 18–24], entering the adult world/early adulthood [2 = 25–34], settling down [3 = 35–44], and middle/late adulthood [4 = 45–70], academic degree (1 = undergraduate; 2 = bachelor; 3 = master and PhD), marital status (1 = single; 2 = married), and children (0 = no; 1 = yes).

*Structural variables* included type of company (0 = public; 1 = private), company size (1 = small 1–25; 2 = medium <250; 3 = large ≥250), and employment status (1 = full-time; 2 = part-time).

*Behavioral variables* were measured by using the questionnaire developed by Sullivan and Mainiero (2008). The questionnaire comprises 15 items (5 items per subscale: authenticity; balance; and challenge) (e.g., “Please indicate the extent to which each of the following statements describes you: *I hunger for greater spiritual growth in my life; I constantly arrange my work around my family needs*; *I continually look for new challenges in everything I do*”). These items consist of a 5-point Likert-type scale (1 = this does not describe me at all, to 5 = this describes me very well). We tested the general factoriality of the scale and confirmed the organization of the items in 3 subscales, explaining 57.56% of the variance. The internal consistency of this scale was assessed through Cronbach’s alpha (α = 0.83) for the total scale (authenticity = 0.74; balance = 0.81; challenge = 0.85).

*Objective success* was measured through one item: the participant’s self-reported current average net salary.

*Subjective success* was measured by using the questionnaire developed by Briscoe et al. (2021). The questionnaire comprises 20 items about the importance and 20 items about the achievement of the following six dimensions: learning and development; work-life balance; positive impact; positive work relationships; financial security; and financial success, each using a Likert scale of 5 points (importance: 1 = not at all important; 5 = extremely important; achievement: 1 = strongly disagree; 5 = strongly agree). The scores of importance and achievement in the six dimensions were computed and weighted in a final score of subjective success. Likert-type scale (1 = this does not describe me at all, to 5 = this describes me very well). The internal consistency of this scale was assessed through Cronbach’s alpha (*α* = 0.92) for the total scale.

### Data collection and analysis procedure

This study is part of a wider project funded by Portuguese national funds through FCT – Fundação para a Ciência e Tecnologia, I.P. under the EXPL/PSI-GER/0321/2021 project, EURECA: New Career Strategies for the New European Remote Careers. Its main objective is to analyze the nature, causes and consequences of the use of strategic career management behaviors in a European sample of remote workers, in order to develop a specific career management model for European adults (aged 18+) who undertake remote work and seek to progress in their chosen career. The project was reviewed and approved by the CRC-W (Catholic Research Centre for Psychological, Family and Social Wellbeing) Review Board. The assessment protocol was developed in English and translated to Portuguese, Spanish, Italian, French and German. Participants were informed of all ethical procedures and data were collected on an online platform (Prolific) in June 2022. Completing the assessment protocol took on average 10 min.^1^ Participants received a financial compensation of 2£ (approximately 2.34€) for their participation.

Descriptive statistics were computed to examine the overall success levels across the different groups of participants. Correlational analysis between all variables were also computed. Hierarchical regression was used to examine the relationship between the predictors and the career success measures. The personal and family variables (gender, age, academic degree, marital status and children) were entered into the analysis in the first step (hypotheses 1 to 5), the structural variables (company type, size and employment status) were entered into the analysis in the second step (hypotheses 6 to 8b), and behavioral variables (strategic career behaviors of authenticity, balance and challenge) were entered into the analysis in the third step (hypotheses 9a-c). All results were considered statistically significant if *p* < 0.05.

## Results

Means, standard deviations, and correlations of all variables used in the analyses and alpha reliabilities of the scales are presented in Table 1.

**Table 1:** Means, standard deviation, correlations, and scale reliabilities.

Variable	*M* (SD)	1	2	3	4	5	6	7	8	9	10	11	12	13
1. Objective success	1177.95 (835.23)													
2. Subjective success	−6.897 (12.29)	0.209***	(0.92)											
3. Gender		−0.061	−0.104**											
4. Age		0.323***	0.077*	0.008										
5. Education		0.291***	0.067	0.110**	0.330***									
6. Marital status		0.276***	0.111**	−0.010	0.429***	0.131***								
7. Children		0.298***	0.136***	0.044	0.531***	0.148***	0.466***							
8. Company type		0.022	0.003	0.151***	−0.081*	−0.081*	−0.013	−0.004						
9. Company size		0.222***	0.057	−0.033	0.214***	0.224***	0.167***	0.136***	−0.187***					
10. Employment status		−0.353***	−0.062	0.068	−0.328***	−0.295***	−240***	−0.207***	0.012	−0.348***				
11. Authenticity	17.98 (4.05)	−0.177**	−0.160***	0.076*	−0.159***	−0.096**	−0.109**	−0.095**	−0.020	−0.059	0.163***	(0.74)		
12. Balance	17.08 (4.27)	0.014	−0.133***	0.045	0.112**	0.013	0.150***	0.196***	−0.038	0.047	−0.029	0.308***	(0.81)	
13. Challenge	15.61 (4.33)	0.125***	0.076*	−0.064	−0.020	0.127***	0.000	0.017	0.003	0.011	−0.012	0.415***	0.182***	(0.85)

Values in parentheses are reliability estimates. ***p < 0.001; **p < 0.01; *p < 0.05.

The results of the hierarchical regression analysis for objective and subjective career success are presented in Table 2 (objective success) and Table 3 (subjective success), respectively.

**Table 2:** Objective success: hierarchical regression results.

Variable and statistic	Standardized betas		
	Step 1	Step 2	Step 3
Step 1. Individual variables			
Gender	−0.073	−0.049	−0.027
Age	0.128*	−0.081	0.087
Education	0.204***	0.162***	0.132**
Marital status	0.134**	0.104***	0.106*
Children	0.135**	0.135**	0.145**
Step 2. Structural variables			
Company type		0.057	0.052
Company size		0.068	0.073
Employment status		−0.173***	−0.171***
Step 3. Behavioral variables			
Authenticity			−0.060
Balance			−0.083*
Challenge			0.192**
*N*	567	567	567
*F*	24.683***	19.268***	15.521***
*R^2^*	0.180	0.216	0.235
Adjusted *R*^2^	0.173	0.205	0.220
Δ *R*^2^		0.032	0.015

Statistically significant results are highlighted in bold. ***p < 0.001; **p < 0.01; *p < 0.05.

**Table 3:** Subjective success: hierarchical regression results.

Variable and statistic	Standardized betas		
	Step 1	Step 2	Step 3
Step 1. Individual variables			
Gender	−0.124**	−0.129**	−0.094*
Age	−0.049	−0.040	−0.041
Education	0.039	0.047	0.009
Marital status	0.057	0.062	0.065
Children	0.135**	0.135**	0.153**
Step 2. Structural variables			
Company type		−0.008	−0.011
Company size		0.010	0.021
Employment status		0.048	0.061
Step 3. Behavioral variables			
Authenticity			−0.159***
Balance			−0.145***
Challenge			0.132**
*N*	596	596	596
*F*	4.564***	2.986**	5.288***
*R^2^*	0.037	0.039	0.091
Adjusted *R*^2^	0.029	0.026	0.073
Δ *R*^2^		0.002	0.051

Statistically significant results are highlighted in bold. ***p < 0.001; **p < 0.01; *p < 0.05.

### Objective success

The “Step 1” column of Table 2 shows that personal and family variables significantly predict objective success outcome (*R^2^* = 0.180, adj. *R^2^* = 0.173, *F*[5, 562] = 24.683, *p* < 0.001), with education being the strongest predictor (*β* = 0.204, *p* < 0.001), marital status and having children being equally strong predictors (*β* = 0.134, *p* = 0.003; *β* = 0.135, *p* = 0.006, respectively), age being the weakest predictor (*β* = 0.128, *p* = 0.011), and gender not being a significant predictor (*β* = −0.073, *p* = 0.058).

The “Step 2” column shows that the model remains significant when structural variables are added, with a slight increase in the variance explained (*R^2^* = 0.216, adj. *R^2^* = 0.205, ∆*R^2^* = 0.032, *F*[8, 559] = 19.268, *p* < 0.001), though employment status is the only significant predictor (*β* = −0.173, *p* < 0.001; the relationship is negative as full-time is coded as =1 and part-time as =2), with type of company and company size falling short of significance (*β* = 0.057, *p* = 0.145; *β* = 0.068, *p* = 0.105, respectively). Finally, the “Step 3” column shows that the model still remains significant when behavioral variables are added, though with a slightly smaller increase in variance explained (*R^2^* = 0.235, adj. *R^2^* = 0.220, ∆*R^2^* = 0.015, *F*[11, 556] = 15.521, *p* < 0.001), with challenge being the strongest predictor (*β* = 0.192, *p* = 0.002), balance being a mild negative predictor (*β* = −0.083, *p* = 0.042), and authenticity not being a significant predictor (*β* = −0.060, *p* = 0.177).

## Subjective success

The “Step 1” column of Table 3 shows that individual variables are a weak but significant predictor (*R^2^* = 0.037, adj. *R^2^* = 0.029, *F*[5, 590] = 4.564, *p* < 0.001), with having children and gender being the strongest predictors (*β* = 0.135, *p* = 0.008; *β* = −0.124, *p* = 0.002, respectively), and age, education and marital status not being significant predictors (*β* = −0.049, *p* = 0.355; *β* = 0.039, *p* = 0.367; *β* = 0.057, *p* = 0.222, respectively). The effect of age on subjective career success did not achieve significance when those aged less than 45 (*M* = 26.12, SD = 5.922, *N* = 695, *R^2^* = 0.000, adj. *R^2^* = −0.001, *F*[1, 693] = 0.012, *p* = 0.913) were compared with those aged over 45 (*M* = 51.75, SD = 5.875, *N* = 44, *R^2^* = 0.039, adj. *R^2^* = 0.016, *F*[1, 42] = 1.694, *p* = 0.200).

Adding structural variables only insignificantly increased variance explained (∆*R^2^* = 0.002, *p* = 0.767) and reduced significance overall (*R^2^* = 0.039, adj. *R^2^* = 0.026, *F*[8, 587] = 2.986, *p* = 0.003), and none of the structural variables were individually significant predictors (company type: *β* = −0.008, *p* = 0.843; company size: *β* = 0.010, *p* = 0.831; employment status: *β* = −0.048, *p* = 0.301). Finally, adding behavioral variables significantly increased variance explained (∆*R^2^* = 0.051, *p* < 0.001) and improved the model’s significance (*R^2^* = 0.091, adj. *R^2^* = 0.073, *F*[11, 584] = 5.288, *p* < 0.001), with authenticity and balance being strong, significant, yet negative, predictors (*β* = −0.159, *p* < 0.001; *β* = −0.145, *p* < 0.001, respectively), and challenge being a mild positive predictor (*β* = 0.132, *p* = 0.003).

## Discussion

This study investigated the effects of a set of individual, structural and behavioral determinants on the objective and subjective career success of a sample of European hybrid workers.

The personal and family factors considered in this study have significant impacts on career success, particularly objective success, which is consistent with previous studies (Ng et al., 2005; Mohd Rasdi et al., 2011). Being male had a positive impact on subjective career success, and being educated had a positive impact on objective career success (*H1*), but education was not a significant predictor of subjective career success and the effect of education on objective career success was irrespective of being male or female.

Furthermore, being female, regardless of education, did have a significant negative impact on subjective career success but not objective career success (*H2*). The lack of gender differences in salary increase/decrease may indicate an improvement among companies toward greater pay equity (Ng et al., 2005). However, there are gender differences in achieving promotions, and some authors suggest that this may be a result of women self-excluding from promotion opportunities in proportion to their willingness and/or necessity to dedicate themselves to family (Ng et al., 2005). A higher education level provides equivalent opportunities for career success between genders but given that women are often associated with jobs in areas which typically have fewer opportunities for objective success, this factor is particularly relevant for men.

*H3* received only weak support, as age was a weak predictor of objective career success, and it was not a significant predictor of subjective career success, and although the strength improved among those aged over 45, the value remained insignificant. Surprisingly, and contrary to previous studies (Lawrence, 1996; Lortie-Lussier and Rinfret, 2005; Cabrera, 2007), the results did not provide strong support for an effect of age on career success measures. However, this lack of support may be due to the relatively low mean age of the participants (27.64 years) with 46.4% under 25, 83.6% under 35 and only 6% aged 45 or over. Effects of age on career success have been shown to occur at later ages (Lawrence, 1996), this would make sense considering that various objective and subjective measures of success take time to achieve or realize. This may also potentially be supported by the increase in predictive value of age when considering only those 45 and over. Although the finding was not significant, this may have been due to the small number of participants (*N* = 44) in this category.

*H4* is mostly not supported, in that being a single male did not have a significant positive impact on objective career success, and while being a male had a positive impact on subjective career success, that impact was irrespective of being married or single. *H5* was not supported, as having children had a significant positive impact on both objective and subjective career success. Results regarding the differences in marital status and children suggest that being partnered and having children may be signs of stability, responsibility, and commitment, regardless of gender (Ng et al., 2005). However, these variables mainly favor men, and more so if they have stay-at-home partners (Lortie-Lussier and Rinfret, 2005). This result is particularly interesting since this is a sample of remote workers for whom having children (especially young children) could affect their capacity for professional dedication and performance.

In regards to structural variables, *H6* and *H7* were not supported, as neither company size nor type were significant predictors of objective or subjective career success. The results for the impact of company size and type fell in line with previous studies that have tended to either show these variables as having, at most, indirect effects on career outcomes (e.g., Kalleberg and Van Buren, 1996; Fernando and Cohen, 2013), or shown mixed results (e.g., Cox and Harquail, 1991; Cox and Nkomo, 1991; Judge et al., 1995; Bozionelos, 1996; Wagner, 1997; Nabi, 1999; Ng et al., 2005; Anderson and Pontusson, 2007; Orser and Leck, 2010; Abele et al., 2011; Al-Hussami et al., 2018; Yeh et al., 2018). Both *H8a* and *H8b* were supported in that employment status was a significant predictor of objective career success (in favor of working full-time) but not a significant predictor of subjective career success. However, the results did show support for working status being a predictor of objective career success, in favor of working full-time. This link should be expected considering that full-time workers work more hours and hence would on average make higher salaries and more quickly gain the experience required for promotions (see also, Nicholson, 2000; Heslin, 2005). The lack of a link between employment status and subjective career success is in line with previous studies (Lee and Johnson, 1991) and may be due in part to self-selection. To the extent that being in either position is a matter of choice, one would expect to see very little difference in levels of subjective career success between the two, as both types of position may provide specific benefits, such as free time to spend on meaningful projects or raising a family in the case of part-time, or financial stability and further opportunities to develop career skills in the case of full-time.

*H9a* received strong support as authenticity was not a significant predictor of objective career success, but was a strong, negative predictor of subjective career success; *H9b* received partial support as balance was a mild, negative predictor of objective career success, but, contrary to the hypothesis, *was* a strong predictor of subjective career success, in the negative direction; and *H9c* received mild support as challenge was a significant predictor of objective career success, but only a mild predictor of subjective career success.

The results of this study on the impact of the KCM behaviors largely support those found by Simmons et al. (2022). Authenticity in the present study was not a significant predictor of objective career success but was a strong, negative predictor of subjective career success, both in line with the previous study. As Simmons et al. (2022) suggest, as an individual puts more emphasis on authenticity, they may come to realize that their current career or status fails to align with their personal values or identity. Furthermore, the situation they find themselves in at this transition may be difficult to redirect or navigate out of, especially if it might entail significant sacrifices to objective career success, or if there are major gaps between their current expertise and that required to pursue more meaningful career goals.

While balance in the present study was a mild, negative predictor of objective career success (salary), in line with the finding of Simmons et al. (2022) that balance significantly predicted salary, balance was, contrary to that study, a strong, negative predictor of subjective career success. These findings may be explained in a similar way as that suggested by Simmons et al. (2022) for authenticity. The efforts made by an individual to achieve balance may, in initial instances and in the short-run, cause detriments to their income and lead to frustrations as they attempt to make the necessary adjustments in their work and non-work schedules, especially if this balance is sought out after an unexpected change.

Challenge in the present study was in contrast to Simmons et al. (2022), who did not find challenge to significantly predict any career outcomes. However, they did find changes in challenge over time to significantly predict salary and career satisfaction, which is in line with the present finding of challenge being a significant predictor of objective career success (salary) and a mild predictor of subjective career success. As Simmons et al. (2022) suggest, a focus on challenge leads an individual to seek out opportunities for career advancement and ways to boost skills and employability, likely providing that individual with strong feelings of pride and achievement and hence greater satisfaction, explaining the link to subjective career success, and leading to the greater skill set, competence and employability that are required for increased salary.

All of these findings point to the importance of investigating changes in KCM behaviors over time, as many of their effects may only become apparent over very long periods of career and personal development.

Table 4 presents a summary of the hypotheses and respective testing results.

**Table 4:** Hypotheses testing: summary.

Determinants	Variables	Hypotheses	Results
Personal variables	Gender and academic degree	H1: Being male and having an academic degree will have a positive impact on achieving career success, particularly objective success. H2: Being female (even if with an academic degree), will have a negative impact on achieving career success (both objective and subjective).	Partially accepted Partially accepted
	Age	H3a: Age will have a positive effect on objective career success. H3b: The strength of the effect of age on subjective career success will lessen after age 45 in this sample of workers.	Weakly accepted Rejected
Family variables	Marital status and children	H4: Being a single man will have a positive impact on achieving career success, particularly objective success. H5: Having children will have a negative impact on achieving objective and subjective career success.	Rejected Rejected
Structural variables	Company size	H6: There will be a positive relationship between company size and both objective measures and subjective of career success.	Rejected
	Company type	H7a: There will be a positive relationship between working in the private sector and objective career success measures. H7b: Working in public or private organizations will not affect differently the subjective career success.	Rejected Rejected
	Employment status	H8a: Part-time workers will score lower on measures of objective career success than full-time workers. H8b: Working part-or full-time will not affect differently the subjective career success.	Partially accepted Partially accepted
Behavioral variables	Strategic career behaviors	H9a: Authenticity will not be a predictor of objective career success, but a negative predictor of subjective career success. H9b: Balance will negatively predict objective career success, but will not be a predictor of subjective career success. H9c: Challenge will positively predict both objective and subjective career success.	Partially accepted Partially accepted Partially accepted

## Limitations

In terms of limitations, because the sample of this research contains workers in different time/week remotely modalities, we can consider these results limited to hybrid workers and not generalizable to all kind of workers after the pandemic. More studies are needed in exploring the difference between remote and traditional workers in strategic career behaviors. It is also important to acknowledge that the sample in this study is younger than the general population for remote or hybrid workers, potentially due to the fact that we used a particular online platform and a financial compensation, which may have affected the selection of respondents. Besides being younger, the respondents had also only had a short experience in remote working, so the main impact of hybrid/remote working in their career may be better observed only in the future.

A second limitation concerns the factoriality of the assessment instruments as well as their levels of internal consistency. We considered these results as a whole, and did not analyze them by questionnaire language. This was also because we had versions of the questionnaires with a small number of participants (e.g., Italian 10.1%, German 0.7%; French 0.7%). Moreover, although the sample was 32% Portuguese, only 27% chose to answer in their mother tongue. For this reason, at this stage we were unable to present any other type of data on the instruments in question.

## Implications and conclusions

Most prior research on career success has assumed traditional career trajectories in their analyses, even though now, especially after the pandemic, careers do not follow this model. The pandemic precipitated the adoption of remote and hybrid work in the organizational world. Up until that time, many companies (mainly from Central and Southern Europe) looked with mistrust at these ways of working. However, presently, there is an increasing number of companies that, after the pandemic, are opting for these new ways of working (Bartik et al., 2020). Nevertheless, it is important to look carefully at the consequences of this decision.

Our study makes significant contributions to the extant literature, research and practice on hybrid workers’ careers, specifically regarding the importance of having a company’s key decision-makers know about the impact that different individual, structural and behavioral variables can have on their employees’ success. This knowledge can prevent a “pre-determination” of opportunities for development, progress and career success based on certain characteristics of employees. Although these results are not so different from those obtained in studies with workers in traditional contexts, it is important to consider that remote or hybrid workers have a unique and important factor that can affect their careers: they are “away from the office,” and hence not seen as regularly, and, for this reason, are more easily forgotten when it comes to opportunities for career success, potentially leading remote and hybrid workers to feel overlooked by their employers when it comes to career advancement opportunities. It is therefore important that companies support their employees, particularly those who work at a distance, in developing strategic career behaviors, that is, behaviors that allow them to manage their careers effectively, consciously, and intentionally in order to be able to achieve their goals. It should be a task for human resources departments to provide short training courses and workshops in which these employees can, on the one hand, understand that because they are away from the office, their perceptions of authenticity, balance and challenge in the work context may be misaligned and, on the other hand, that there is a set of strategic behaviors that can be applied to balance these perceptions and thus ensure adequate levels of success both objectively and subjectively. Constant monitoring by companies of these perceptions of their employees, particularly those in hybrid or remote situations, can also encourage preventive and promotional action rather than remedial action.

It is therefore important to contrast the different career developments of workers in different work situations (traditional vs. remote vs. hybrid) and the impact these variables can have on workers’ levels of success, satisfaction, and well-being with their careers over time. It is also important to move forward with studies that determine which criteria managers do and should take into account when considering an employee for career advancement opportunities, and whether they are aware of the impact that these variables can have on their decisions. There are also other variables that can impact both objective and subjective career success, such as, among others, a company’s organizational culture and a company’s (objective or perceived) reputation, which need to be investigated as a future line of research. In this study, we addressed some of the factors that should be considered in the career success of workers in order to encourage development-oriented policies that support HR departments and employees, because workers with clear, specific, realistic career goals and action plans that are aligned with their organization’s strategy are also more motivated, engaged and productive workers.

Due to its exploratory nature, the obtained results illuminate the importance of considering and continuing to study the perception of workers in hybrid and remote modality.

## Data availability statement

The raw data supporting the conclusions of this article will be made available by the authors, without undue reservation.

## Ethics statement

The studies involving human participants were reviewed and approved by Católica Research Centre for Psychological, Family and Social Wellbeing. The patients/participants provided their written informed consent to participate in this study.

## Author contributions

JP substantial contribution to the concept and design of the study and of the manuscript, oversight and leadership responsibility for the research activity planning and execution, and literature review. KH: literature review, statistical analysis, and manuscript first draft. MP review of the statistical analysis and data interpretation. SC critically review of the manuscript for important intellectual content, namely on discussion, conclusion, and implications. All authors contributed to the article and approved the submitted version.

## Funding

This work is financed by Portuguese national funds through the FCT – Foundation for Science and Technology, I.P. under project EXPL/PSI-GER/0321/2021 (EURECA: New career strategies for the new European remote careers).

## Conflict of interest

The authors declare that the research was conducted in the absence of any commercial or financial relationships that could be construed as a potential conflict of interest.

## Publisher’s note

All claims expressed in this article are solely those of the authors and do not necessarily represent those of their affiliated organizations, or those of the publisher, the editors and the reviewers. Any product that may be evaluated in this article, or claim that may be made by its manufacturer, is not guaranteed or endorsed by the publisher.

## Footnotes

^1^Link to the protocol: https://ucpcienciashumanas.eu.qualtrics.com/jfe/form/SV_egQUJXlNwiGwQPs.
